# Nationwide Availability of and Enrollment in Medicare and Medicaid Dual-Eligible Special Needs Plans With Exclusively Aligned Enrollment

**DOI:** 10.1001/jamahealthforum.2024.3546

**Published:** 2024-10-18

**Authors:** Kenton J. Johnston, Michelle Hendricks, Megha Dabas, Eliza Macneal, Jeah Jung, David J. Meyers, Jose F. Figueroa, Eric T. Roberts

**Affiliations:** 1 General Medical Sciences Division, School of Medicine, Washington University in St Louis, St Louis, Missouri; 2 Leonard Davis Institute of Health Economics, University of Pennsylvania, Philadelphia; 3 College of Public Health, George Mason University, Fairfax, Virginia; 4 School of Public Health, Brown University, Providence, Rhode Island; 5 T. H. Chan School of Public Health, Harvard University, Boston, Massachusetts; 6 Perelman School of Medicine, University of Pennsylvania, Philadelphia

## Abstract

**Importance:**

In 2021, the Centers for Medicare & Medicaid Services designated a new category of dual-eligible special needs plans (D-SNPs) with exclusively aligned enrollment (receive Medicare and Medicaid benefits through the same plan or affiliated plans within the same organization).

**Objective:**

To assess the availability of and enrollment in D-SNPs with exclusively aligned enrollment and to compare the characteristics of beneficiaries enrolled in D-SNPs with exclusively aligned enrollment available vs beneficiaries without such enrollment available.

**Design, Setting, and Participants:**

Full-benefit beneficiaries enrolled in D-SNPs for 6 months or longer in 2021 or 2022. Availability of and beneficiary enrollment in D-SNPs were assessed by year and county for D-SNPs with exclusively aligned enrollment available vs D-SNPs without exclusively aligned enrollment available. The D-SNP enrollees residing in counties with aligned plans available were compared based on demographic, social, health, and area characteristics vs D-SNP enrollees in counties without such plans available. Comparisons were also made based on beneficiaries who enrolled in the aligned D-SNPs vs those who did not enroll (were enrolled in unaligned D-SNPs). The data analyses were conducted from October 1, 2023, to August 2, 2024.

**Main Outcomes and Measures:**

Availability of aligned D-SNPs and beneficiary residence by county; enrollment in exclusively aligned D-SNPs vs unaligned D-SNPs; and beneficiary demographic, social, health, and area characteristics.

**Results:**

Of 2 197 732 beneficiaries enrolled in D-SNPs in 2021, 881 736 (40.1%) were living in counties with aligned enrollment available and 251 305 (11.4%) enrolled. Of 2 689 045 beneficiaries enrolled in D-SNPs in 2022, 1 047 223 (38.9%) were living in counties with aligned enrollment available and 318 906 (11.9%) enrolled. Beneficiaries enrolled in D-SNPs residing in counties without aligned enrollment available were more likely to live in rural or micropolitan areas (21.9%) vs beneficiaries in counties with aligned enrollment available (8.1%) (standardized mean difference [SMD], 0.38 [95% CI, 0.38-0.38]), be entitled to disability (44.4% vs 27.3%, respectively; SMD, 0.36 [95% CI, 0.36-0.36]), or be Black individuals (27.4% vs 21.4%; SMD, 0.14 [95% CI, 0.14-0.14]); were less likely to be Hispanic individuals (15.4% vs 33.7%; SMD, 0.45 [95% CI, 0.45-0.45]) or Asian or Pacific Islander individuals (6.1% vs 12.2%; SMD, 0.22 [95% CI, 0.22-0.22]); and lived in zip codes with a higher area deprivation index (mean, 66.8 [SD, 26.4] vs mean, 43.2 [SD, 29.0]; SMD, 0.86 [95% CI, 0.86-0.86]). Beneficiaries enrolled in aligned D-SNPs were more likely to be receiving long-term institutionalized care vs beneficiaries in nonaligned D-SNPs (4.3% vs 1.0%, respectively; SMD, 0.24 [95% CI, 0.24-0.25]) or have dementia or Alzheimer disease (9.2% vs 5.9%; SMD, 0.13 [95% CI, 0.13-0.13]).

**Conclusions:**

This study found that availability of and enrollment in D-SNPs with exclusively aligned enrollment are increasing, but the overall proportion enrolled remains low. Further reforms are needed to promote aligned enrollment.

## Key Points

**Question:**

To what extent are dual-eligible special needs plans (D-SNPs) with exclusively aligned enrollment (receive Medicare and Medicaid benefits through the same plan or affiliated plans within the same organization) available and what is the uptake of such plans nationally?

**Findings:**

In this cohort study of full-benefit enrollees in D-SNPs, 40.1% of 2.2 million beneficiaries resided in counties with exclusively aligned enrollment available in 2021 and 11.4% enrolled; in 2022, 38.9% of 2.7 million beneficiaries resided in counties with exclusively aligned enrollment available and 11.9% enrolled, which is a slight increase from 2021.

**Meaning:**

The availability of and enrollment in D-SNPs with exclusively aligned enrollment are increasing, but the overall proportion of enrolled beneficiaries remains low.

## Teaser

This national cohort study assesses the availability of and enrollment in dual-eligible special needs plans among dual-eligible beneficiaries from 2021 to 2022.

## Introduction

The 12.8 million dual-eligible beneficiaries enrolled in both Medicare and Medicaid are among the most medically and socially vulnerable patients in the US.^1,2^ For such patients, coordination of medical care, social and behavioral health services, and long-term care are important for meeting care needs and improving quality of life.^3,4,5,6^ However, because Medicare and Medicaid are provided through separate and uncoordinated programs, dual-eligible beneficiaries are susceptible to receiving fragmented and inefficient care.^5,7^

Therefore, policymakers are pursuing strategies to integrate coverage for dual-eligible beneficiaries in managed care plans that coordinate care across Medicare and Medicaid.^5,8,9,10^ Medicare Advantage dual-eligible special needs plans (D-SNPs) have emerged as a key platform for integration,^9^ and more than 3.3 million dual-eligible beneficiaries with full Medicaid are enrolled in such plans.^11^ The D-SNPs exist on a continuum of integration from (1) coordination only, which offer minimal integration with Medicaid, (2) highly integrated, which contract with state Medicaid agencies to cover long-term services or behavioral health services or both types of services, and (3) fully integrated, which integrate most or all Medicaid services with Medicare.^9,12^

Providing Medicare and Medicaid coverage through a single managed care plan or organization is considered by many policymakers to be an important prerequisite for integrating care.^12^ Despite widespread use of managed care in Medicaid, and recent use of integrated care plans (such as D-SNPs), systematic reviews suggest the evidence is mixed regarding the effectiveness of managed care and integrated plans in improving patient care and outcomes and reducing costs; however, perceived effectiveness has been more positive for fully integrated D-SNPs, which have the highest degree of integration.^13,14,15^

Many D-SNPs enroll individuals whose Medicaid coverage is provided through a separate plan or parent organization.^9^ This lack of aligned enrollment is a concern because policymakers believe that it may diminish opportunities for D-SNPs to coordinate and manage care for beneficiaries of Medicare and Medicaid. As a result, in 2021, the Centers for Medicare & Medicaid Services (CMS) designated a new category of D-SNPs with exclusively aligned enrollment, referred to by the CMS as applicable integrated plans (hereafter referred to as “aligned” or “exclusively aligned” plans). Within aligned D-SNPs, all enrollees must receive Medicare and Medicaid benefits exclusively through the same plan or affiliated plans within the same organization.^9,16^ The D-SNPs that receive this aligned designation are able to coordinate benefits and simplify administrative processes by using unified procedures for appeals and grievances.^17,18^

Furthermore, the CMS issued new rules in 2022 requiring all fully integrated D-SNPs to operate with exclusively aligned enrollment by 2025.^9,19^ These policies have the potential to shape access to and enrollment in integrated plans, particularly because many dual-eligible beneficiaries are expected to transition into D-SNPs after the unwinding of integrated Medicare and Medicaid plans under the CMS financial alignment initiative.^20^ Consequently, aligned D-SNPs are likely to become the predominant model for integration of Medicare and Medicaid coverage.

This study focused on the following questions. First, how many beneficiaries live in counties with exclusively aligned D-SNPs available? Second, how do these beneficiaries compare with beneficiaries who live in counties without aligned D-SNPs available? Third, among beneficiaries enrolled in D-SNPs and residing in counties with aligned plans available, how many enroll in aligned D-SNPs? Fourth, how do the beneficiaries enrolled in aligned D-SNPs compare with beneficiaries who do not enroll?

## Methods

This study was approved by the institutional review board of Washington University in St Louis. Informed consent was waived because the information used involved no more than a minimal risk to privacy and the research could not be conducted without access to and use of the requested information. The data analyses were conducted from October 1, 2023, to August 2, 2024. The study followed the Strengthening the Reporting of Observational Studies in Epidemiology (STROBE) reporting guideline for cohort studies.

### Data and Study Population

We conducted a national cohort study of full-benefit dual-eligible beneficiaries enrolled in D-SNPs during 2021 and 2022. The analyses used 100% Master Beneficiary Summary File enrollment data from the CMS Chronic Condition Data Warehouse for 2020, 2021, and 2022 as well as 100% Medicare fee-for-service claims and Medicare Advantage encounter data for 2020 and 2021. Beneficiaries were included if they were enrolled for 6 months or longer in a full-benefit D-SNP in 2021 or 2022 (or had continuous enrollment until death) plus a full prior baseline year of continuous Medicare enrollment (2020 for beneficiaries of 2021 D-SNPs and 2021 for beneficiaries of 2022 D-SNPs).

Although no coordination-only D-SNPs were designated as exclusively aligned in 2021 or 2022, we included beneficiaries enrolled in these plans (in addition to beneficiaries enrolled in highly integrated D-SNPs and fully integrated D-SNPs) in the study population because most beneficiaries in coordination-only SNPs who resided in counties where aligned D-SNPs were offered were eligible to enroll if they wanted to during the study period. Furthermore, all beneficiaries of D-SNPs are the target of alignment efforts, and, beginning in 2023, some coordination-only D-SNPs have been designated as exclusively aligned.

Beneficiaries were excluded if they (1) resided outside the 50 US states or the District of Columbia, (2) had end-stage kidney disease, (3) had missing geographic data, (4) were living in counties without D-SNPs available, or (5) lacked evidence of residence in a county with aligned D-SNPs despite enrollment in aligned D-SNPs (0.3% of such enrollees). The unit of analysis was the beneficiary-year.

### Availability and Uptake of D-SNPs by County

The CMS data on D-SNPs were linked to data identifying (1) exclusively aligned plans, (2) counties with D-SNPs and aligned D-SNPs available, (3) beneficiary county of residence, and (4) plan enrollment data in the Master Beneficiary Summary File.^21,22,23^ We then identified beneficiary enrollment in plans that were designated by the CMS as exclusively aligned D-SNPs in 2021 and 2022 vs those enrolled in D-SNPs that were not designated as aligned (unaligned). Beneficiary enrollment months and current Medicare entitlement status were also assessed.

### Prior-Year Plan and Beneficiary Characteristics

To assess previous sources of Medicare coverage among D-SNP enrollees living in counties with aligned plans available, we linked prior-year Master Beneficiary Summary File records (from 2020 and 2021) identifying enrollment in traditional Medicare, Programs of All-Inclusive Care for the Elderly, integrated Medicare and Medicaid plans, coordination-only D-SNPs, fully integrated D-SNPs, highly integrated D-SNPs, and other Medicare Advantage plans.

We further assessed beneficiary demographic, social, health, and area of residence (urban or metropolitan vs rural or micropolitan) characteristics during the baseline years (2020 or 2021 [prior to the study year for each beneficiary-year]) among the entire study population. The characteristics included age, sex, race and ethnicity (using Research Triangle Institute algorithm codes), area deprivation index (based on zip code),^24^ county-level penetration for Medicare Advantage plans, and residence in a rural or micropolitan county (defined by Rural-Urban Continuum Codes).^25^

Major chronic comorbidities were also assessed and included diabetes, heart failure, ischemic heart disease, stroke, chronic obstructive pulmonary disease or asthma, and chronic kidney disease and the neuropsychiatric comorbidities of Alzheimer disease and related dementias, intellectual or developmental disability, depression, anxiety disorder, and serious mental illness (bipolar disorder, schizophrenia, and psychosis) using the CMS Chronic Condition Data Warehouse condition algorithms^26,27^ applied to the baseline years of traditional Medicare claims and Medicare Advantage encounters (after removing Medicare Advantage chart reviews). In addition, we measured long-term institutionalized care using a previously validated algorithm^28^ and the CMS Minimum Data Set 3.0, frailty using a claims-based frailty index,^29^ and risk score for the CMS Hierarchical Condition Categories.^30^

### Statistical Analysis

We identified the number of coordination-only, highly integrated, and fully integrated D-SNPs with vs without exclusively aligned enrollment available and mapped the availability of highly integrated and fully integrated D-SNPs and exclusively aligned plans across counties (no coordination-only D-SNPs were exclusively aligned in 2021 or 2022). Then we compared beneficiaries residing in counties with aligned D-SNPs available in 2021 and 2022 vs beneficiaries residing in counties without aligned D-SNPs available on local area and beneficiary characteristics at baseline. In the sensitivity analyses, we removed health risk assessments from baseline traditional Medicare claims and Medicare Advantage encounters,^31^ along with beneficiary-year observations in Medicare Advantage contracts with a high degree of encounter record missingness by applying a modified version of the approach described by Jung et al,^32^ and then reestimated the comparisons.

To characterize the flow of dual-eligible beneficiaries residing in counties with aligned D-SNPs available across D-SNP types, we created Sankey diagrams depicting the proportion of enrollees in 2021 and 2022 in aligned D-SNPs and unaligned D-SNPs who in December of the prior year were enrolled in traditional Medicare; integrated Medicare and Medicaid plans; Programs of All-Inclusive Care for the Elderly; coordination-only, fully integrated D-SNPs, or highly integrated D-SNPs; and other Medicare Advantage plans. We then analyzed total enrollment in coordination-only, highly integrated, and fully integrated D-SNPs with vs without exclusively aligned enrollment available in 2021 and 2022. In addition, among enrollees residing in counties with aligned D-SNPs available, we compared the baseline characteristics of beneficiaries who enrolled vs those who did not enroll in plans that were designated as exclusively aligned D-SNPs in 2021 and 2022.

We calculated standardized mean differences (SMDs) for continuous and categorical variables, and considered an SMD of less than 0.10 to be negligible.^33,34^ The analyses were conducted using SAS version 9.4 (SAS Institute Inc) and version 4.3.1 of R Studio.

## Results

### Description of Study Population

There were 4 952 506 beneficiary-years with 6 months or longer of continuous full-benefit D-SNP enrollment in 2021 or 2022 plus a prior year of continuous Medicare enrollment (eFigure 1 in Supplement 1). After applying exclusions (1.3%), the final study population comprised 4 886 777 beneficiary-years.

### Aligned Plan Availability and Comparison of Beneficiaries by County of Residence

In 2021, 57 of 69 fully integrated D-SNPs, 15 of 167 highly integrated D-SNPs, and 0 of 338 coordination-only D-SNPs were designated as exclusively aligned D-SNPs (eFigure 2 in Supplement 1). In 2022, 60 of 72 fully integrated D-SNPs, 23 of 199 highly integrated D-SNPs, and 0 of 409 coordination-only D-SNPs were designated as exclusively aligned D-SNPs.

**Figure 1.**
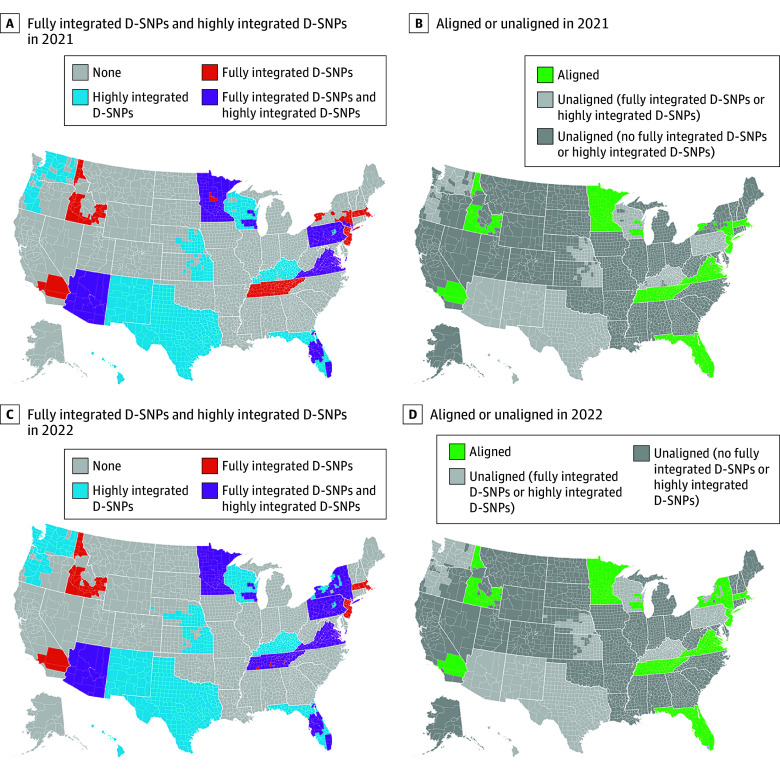
Availability of Exclusively Aligned Fully Integrated or Highly Integrated Dual-Eligible Special Needs Plans (D-SNPs) in US Counties in 2021 and 2022 The maps were created by linking annual (2021 and 2022) D-SNP contract and plan identifiers from the Centers for Medicare & Medicaid Services integration status file, which contains D-SNP integration and alignment status, to the National Bureau of Economic Research landscape files, which identify the counties where the D-SNPs were offered by year, and by linking to the US county map reprojection using SAS (SAS Institute Inc).

In 2021, aligned D-SNPs were available in 489 counties throughout Florida, Minnesota, and Tennessee and in select areas of California, Idaho, Massachusetts, New Jersey, New York, Virginia, and Wisconsin (Figure 1). In 2022, the availability of aligned D-SNPs expanded into 27 additional counties—mainly in upstate New York and Virginia. Due to the unavailability of highly integrated and fully integrated D-SNPs in many counties, and the low number of exclusively aligned D-SNPs (83 of 680 plans in 2022), many dual-eligible beneficiaries did not have the option of enrolling in an aligned D-SNP during 2021 and 2022.

Nearly 4 in 10 beneficiaries (n = 1 928 959 beneficiary-years) resided in counties with aligned D-SNPs available in 2021 or 2022 and more than 6 in 10 beneficiaries (n = 2 957 818) did not reside in counties where aligned D-SNPs were available (Table 1). Beneficiaries living in counties without available aligned D-SNPs were more likely to live in a rural or micropolitan area (21.9%) compared with beneficiaries living in counties with aligned D-SNPs available (8.1%) (SMD, 0.38 [95% CI, 0.38-0.38]), were more likely to live in zip code areas with higher area deprivation index scores (indicating greater area deprivation) (mean, 66.8 [SD, 26.4] vs mean, 43.2 [SD, 29.0], respectively; SMD, 0.86 [95% CI, 0.86-0.86]), were more likely to live in counties with lower average Medicare Advantage penetration rates (45.1% vs 48.2%; SMD, 0.26 [95% CI, 0.26-0.26]), were more likely to receive disability benefits (44.4% vs 27.3%; SMD, 0.36 [95% CI, 0.36-0.36]), were younger in age (mean, 63.6 years [SD, 14.3 years] vs mean, 68.3 years [SD, 13.8 years]; SMD, 0.34 [95% CI, 0.33-0.34]), were more likely to be non-Hispanic White individuals (48.7% vs 29.7%; SMD, 0.40 [95% CI, 0.39-0.40]), and were more likely to be Black individuals (27.4% vs 21.4%; SMD, 0.14 [95% CI, 0.14-0.14]); were less likely to be Hispanic individuals (15.4% vs 33.7%; SMD, 0.45 [95% CI, 0.45-0.45]), were less likely to be Asian or Pacific Islander individuals (6.1% vs 12.2%; SMD, 0.22 [95% CI, 0.22-0.22]), and were less likely to have dementia or Alzheimer disease (4.5% vs 6.8%; SMD, 0.11 [95% CI, 0.10-0.11]); and were more likely to have an anxiety disorder (22.4% vs 18.1%; SMD, 0.11 [95% CI, 0.10-0.11]). In a sensitivity analysis excluding Medicare Advantage contracts with incomplete data and health risk assessments, the results were similar (eTable in Supplement 1).

**Table 1.** Baseline Characteristics of Beneficiaries in Full-Benefit Dual-Eligible Special Needs Plans (D-SNPs) by Residence in County With Aligned D-SNPs Available in 2021 and 2022

Characteristics	Residence in county with aligned D-SNPs available^a^		Standardized mean difference (95 CI)^c^
	No	Yes^b^	
Beneficiaries, No.^d^	1 786 573	1 136 919	NA
Beneficiary-years	2 957 818	1 928 959	NA
Local area			
Area deprivation index score, mean (SD)^e^	66.8 (26.4)	43.2 (29.0)	0.86 (0.86 to 0.86)
Rural or micropolitan (vs urban or metropolitan)	21.9	8.1	0.38 (0.38 to 0.38)
Penetration rate of Medicare Advantage^f^	45.1	48.2	0.26 (0.26 to 0.26)
Total enrollment, mean (SD), mo	11.8 (1.4)	11.8 (1.4)	0 (−0 to 0)
Type of D-SNP^g^			
Highly integrated	26.0	48.6	0.49 (0.49 to 0.49)
Fully integrated	3.6	25.5	0.71 (0.71 to 0.71)
Coordination only	70.4	25.9	0.99 (0.99 to 0.99)
Medicare entitlement status			
Age ≥65 y	55.6	72.7	0.36 (0.36 to 0.36)
Disability	44.4	27.3	0.36 (0.36 to 0.36)
**Demographics**			
Age, mean (SD), y	63.6 (14.3)	68.3 (13.8)	0.34 (0.33 to 0.34)
Sex			
Female	63.0	62.7	0.01 (0 to 0.01)
Male	37.0	37.3	0.01 (0 to 0.01)
Race and ethnicity^h^			
American Indian or Alaska Native	0.8	0.3	0.07 (0.06 to 0.07)
Asian or Pacific Islander	6.1	12.2	0.22 (0.22 to 0.22)
Black	27.4	21.4	0.14 (0.14 to 0.14)
Hispanic	15.4	33.7	0.45 (0.45 to 0.45)
Non-Hispanic White	48.7	29.7	0.40 (0.39 to 0.40)
Other^i^	1.7	2.8	0.08 (0.08 to 0.08)
**Health status**			
Centers for Medicare & Medicaid Services Hierarchical Condition Categories risk score (version 28), mean (SD)^j^	1.6 (1.5)	1.5 (1.4)	0.01 (0.01 to 0.02)
Long-term institutionalized care^k^	1.8	1.9	0.01 (0.01 to 0.01)
Frailty level (for those with a community-dwelling age ≥65 y)^l^			
Robust (lowest level)	33.3	35.7	0.05 (0.05 to 0.05)
Prefrailty	47.6	47.3	0 (−0.01 to 0)
Mild frailty	14.8	13.2	0.05 (0.05 to 0.05)
Moderate to severe frailty	4.4	3.9	0.03 (0.02 to 0.03)
Major chronic comorbidities			
Diabetes	32.6	36.2	0.08 (0.07 to 0.08)
Heart failure	10.1	10.2	0 (0 to 0.01)
Ischemic heart disease	13.3	14.4	0.03 (0.03 to 0.03)
Stroke or transient ischemic attack	3.5	3.9	0.02 (0.01 to 0.02)
Chronic obstructive pulmonary disease or asthma	24.5	22.5	0.05 (0.05 to 0.05)
Chronic kidney disease	15.2	17.9	0.07 (0.07 to 0.07)
Neuropsychiatric comorbidities			
Dementia or Alzheimer disease	4.5	6.8	0.11 (0.10 to 0.11)
Intellectual or developmental disability	3.2	2.1	0.07 (0.07 to 0.07)
Depression	21.5	21.1	0.01 (0.01 to 0.01)
Anxiety disorder	22.4	18.1	0.11 (0.10 to 0.11)
Serious mental illness (bipolar disorder, schizophrenia/psychosis)	12.8	10.1	0.08 (0.08 to 0.08)

Abbreviation: NA, not applicable.

^a^ Data are expressed as mean percentages unless otherwise indicated. Available was defined as a county in which at least 1 aligned D-SNP was operating (identified using Applicable Integrated Plan indicator data).

^b^ Determined based on the county where they were living during the first month of enrollment. If the county was missing, either the county in a subsequent month or the county identified in the Social Security Administration data was used.

^c^ The approach for calculating these data was based on the methods of Austin.^33^

^d^ Included Medicare beneficiaries with at least 1 calendar year (2020 or 2021) of continuous enrollment in Medicare Parts A and B and 1 study year (2021 or 2022) with at least 6 months enrollment in a full benefit D-SNP.

^e^ The score is a percentile ranking (range, 1-100); higher values indicate greater deprivation. The score was missing for 269 722 beneficiary-years (5.5% of the total).

^f^ Based on July of the study year.

^g^ Had at least 6 months of D-SNP enrollment.

^h^ Identified from the Research Triangle Institute (RTI) race code.

^i^ Combined category from the RTI race codes of “other” and “unknown.”

^j^ Missing for 132 beneficiary-years (0.002% of the total).

^k^ Defined as 100 days or longer.

^l^ Data were available for 1 382 617 beneficiary-years.

### Prior-Year Plan and Enrollment in Aligned D-SNPs

**Figure 2.**
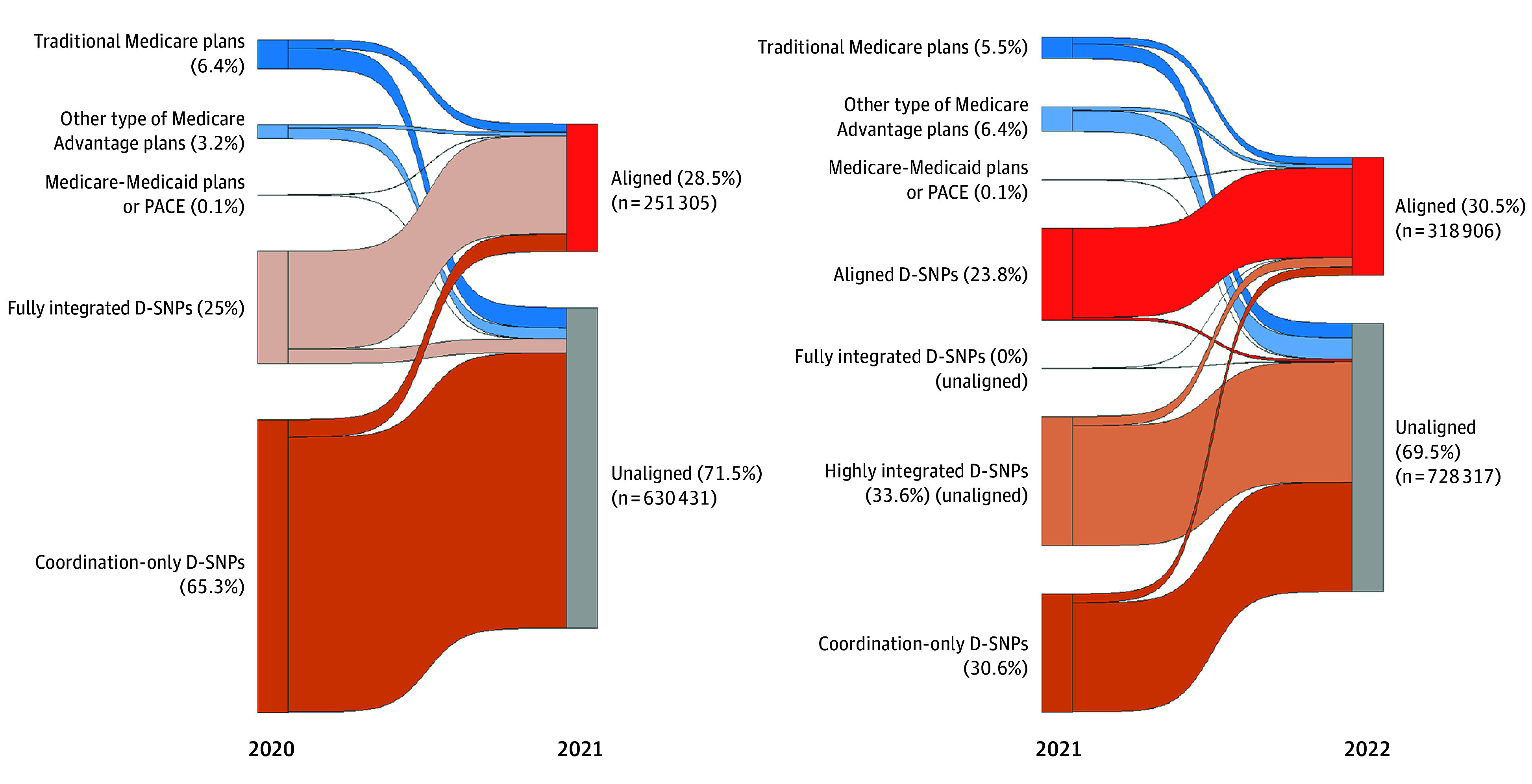
Prior-Year Medicare Plan for Full Benefit Dual-Eligible Beneficiaries Living in Counties With Available Aligned Dual-Eligible Special Needs Plans (D-SNPs) Includes Medicare beneficiaries with (1) at least 1 calendar year (2020 or 2021) of continuous baseline enrollment in Medicare Parts A and B (traditional Medicare or Medicare Advantage), (2) a following year (2021 or 2022) of at least 6 months of full benefit D-SNP enrollment, and (3) residence in a county where an aligned D-SNP was available. PACE indicates Programs of All-Inclusive Care for the Elderly. Beneficiaries were excluded if they had end-stage kidney disease, were living outside the 50 US states and the District of Columbia, and the penetration rate of Medicare Advantage by county or Rural-Urban Continuum Codes was missing. Individuals who died during the first 6 months of the study year were included if they met the criteria up to the month of their death. Beneficiaries were assigned to the plan that they had for the most months during the study year. If 2 plans had the same number of months, then beneficiaries were assigned to the last plan they had during the study year. For the prior year, beneficiaries were assigned to the plan they had in December. The aligned D-SNPs were identified using the Applicable Integrated Plan indicator data published by the Centers for Medicare & Medicaid Services and linked by unique 5-digit contract and 3-digit SNP identifiers using the contract and plan benefit package numbers for Medicare Part C in the Master Beneficiary Summary File. For beneficiaries with a single combination contract number and plan benefit package number operating in 2 states with 2 separate D-SNP types (eg, 1 coordination-only D-SNP and 1 highly integrated D-SNP), the D-SNPs were treated as 2 separate plans.

Among 881 736 beneficiaries residing in counties with aligned D-SNPs available in 2021, 28.5% enrolled (Figure 2). Most enrollees in aligned D-SNPs in 2021 were enrolled in fully integrated D-SNPs in the prior year; however, a small proportion came from coordination-only D-SNPs, other Medicare Advantage plans, or traditional Medicare. Among 1 047 223 beneficiaries residing in counties with aligned D-SNPs available in 2022, 30.5% enrolled. In 2022, most beneficiaries enrolled in aligned D-SNPs were enrolled in aligned D-SNPs during the prior year; however, a substantial minority came from highly integrated D-SNPs, coordination-only D-SNPs, traditional Medicare, and Medicare Advantage plans.

### Total Enrollment in D-SNPs and Aligned D-SNPs

**Figure 3.**
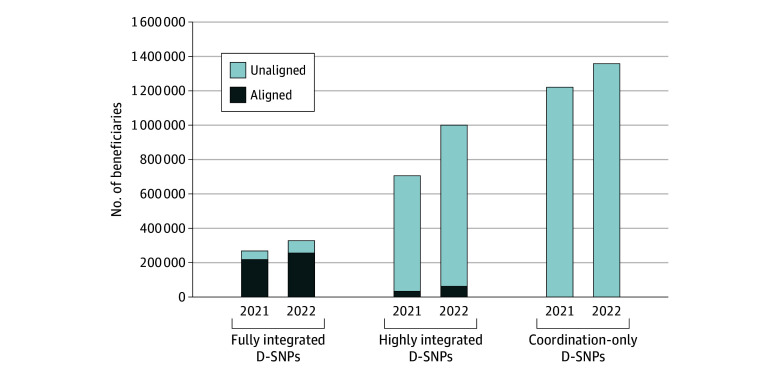
Enrollment in Dual-Eligible Special Needs Plans (D-SNPs) by Plan Type and Alignment Status in 2021 and 2022 Includes Medicare beneficiaries with at least 1 month of full-benefit D-SNP enrollment in 2021 or 2022. Beneficiaries were assigned to the plan that they had for the most months during the study year. If 2 plans had the same number of months, beneficiaries were assigned to the last plan they had during the study year. The aligned D-SNPs were identified using the Applicable Integrated Plan indicator data published by the Centers for Medicare & Medicaid Services and linked by unique 5-digit contract and 3-digit D-SNP identifiers using the contract and plan benefit package numbers for Medicare Part C in the Master Beneficiary Summary File. For beneficiaries with a single combination contract number and plan benefit package number operating in 2 states with 2 separate D-SNP types (eg, 1 coordination-only D-SNP and 1 highly integrated D-SNP), the D-SNPs were treated as 2 separate plans.

Of 2 197 732 full-benefit D-SNP beneficiaries in 2021, 251 305 (11.4%) were enrolled in aligned D-SNPs; in 2022, of 2 689 045 full-benefit D-SNP beneficiaries, 318 906 (11.9%) were enrolled in aligned D-SNPs (Figure 3). In 2021, aligned D-SNP enrollees made up 80.9% of beneficiaries in fully integrated D-SNPs and 4.7% of beneficiaries in highly integrated D-SNPs; in 2022, aligned D-SNP enrollees made up 78.1% of beneficiaries in fully integrated D-SNPs and 6.2% of beneficiaries in highly integrated D-SNPs.

### Comparison of Beneficiaries by Enrollment in Aligned D-SNPs

Among beneficiaries who resided in counties with aligned D-SNPs available, those who did not enroll in aligned D-SNPs were more likely to be enrolled in highly integrated D-SNPs (61.9%) vs beneficiaries who did enroll in aligned D-SNPs (16.9%) (SMD, 0.99 [95% CI, 0.99-0.99]), were less likely to be enrolled in fully integrated D-SNPs (1.3% vs 83.1%, respectively; SMD, 3.64 [95% CI, 3.64-3.65]), were more likely to live in counties with higher mean rates of market penetration for Medicare Advantage (50.7% vs 42.4%; SMD, 0.65 [95% CI, 0.65-0.65]), were more likely to receive disability benefits (29.9% vs 21.3%; SMD, 0.19 [95% CI, 0.19-0.20]), were younger in age (mean, 67.4 years [SD, 13.8 years] vs mean, 70.4 years [SD, 13.6 years]; SMD, 0.22 [95% CI, 0.22-0.22]), were less likely to be non-Hispanic White individuals (27.2% vs 35.5%; SMD, 0.18 [95% CI, 0.18-0.19]), were less likely to receive long-term institutionalized care (1.0% vs 4.3%; SMD, 0.24 [95% CI, 0.24-0.25]), were more likely to be at the lowest level for frailty (37.5% vs 31.7%; SMD, 0.12 [95% CI, 0.12-0.12]), and were less likely to have dementia or Alzheimer disease (5.9% vs 9.2%; SMD, 0.13 [95% CI, 0.13-0.13]) (Table 2). The differences narrowed among the beneficiaries based on rurality; area deprivation index scores; Black, Hispanic, or Asian or Pacific Islander race and ethnicity; and likelihood of having an anxiety disorder (SMD <0.10) vs the comparisons based on residence in counties with vs without aligned D-SNPs available.

**Table 2.** Baseline Characteristics for Beneficiaries in Full-Benefit Dual-Eligible Special Needs Plans (D-SNPs) by Enrollment in Aligned D-SNPs in 2021 and 2022

Characteristics	Enrollment in aligned D-SNPs^a^		Standardized mean difference (95 CI)^c^
	Enrolled^b^	Not enrolled	
Beneficiaries, No.^d^	351 623	835 673	NA
Beneficiary-years	570 211	1 358 748	NA
Local area			
Area deprivation index score, mean (SD)^e^	42.1 (25.3)	43.7 (30.4)	0.06 (0.05-0.06)
Rural or micropolitan (vs urban or metropolitan)	8.6	7.8	0.03 (0.03-0.03)
Penetration rate of Medicare Advantage^f^	42.4	50.7	0.65 (0.65-0.65)
Total enrollment, mean (SD), mo	11.7 (1.6)	11.8 (1.3)	0.07 (0.07-0.08)
Type of D-SNP^g^			
Highly integrated	16.9	61.9	0.99 (0.99-0.99)
Fully integrated	83.1	1.3	3.64 (3.64-3.65)
Coordination only	0	36.8	0.91 (0.91-0.91)
Medicare entitlement status			
Age ≥65 y	78.7	70.1	0.19 (0.19-0.20)
Disability	21.3	29.9	0.19 (0.19-0.20)
**Demographics**			
Age, mean (SD), y	70.4 (13.6)	67.4 (13.8)	0.22 (0.22-0.22)
Sex			
Female	64.5	62.0	0.05 (0.05-0.05)
Male	35.5	38.0	0.05 (0.05-0.05)
Race and ethnicity^h^			
American Indian or Alaska Native	0.4	0.2	0.03 (0.03-0.03)
Asian or Pacific Islander	10.4	12.9	0.08 (0.07-0.08)
Black	19.0	22.4	0.08 (0.08-0.08)
Hispanic	31.2	34.8	0.08 (0.07-0.08)
Non-Hispanic White	35.5	27.2	0.18 (0.18-0.19)
Other^i^	3.5	2.5	0.06 (0.06-0.06)
**Health status**			
Centers for Medicare & Medicaid Services Hierarchical Condition Categories risk score (version 28), mean (SD)^j^	1.6 (1.4)	1.5 (1.4)	0.08 (0.08-0.09)
Long-term institutionalized care^k^	4.3	1.0	0.24 (0.24-0.25)
Frailty level (for those with a community-dwelling age ≥65 y)^l^			
Robust (lowest level)	31.7	37.5	0.12 (0.12-0.12)
Prefrailty	48.0	47.0	0.02 (0.02-0.02)
Mild frailty	15.5	12.1	0.10 (0.10-0.10)
Moderate to severe frailty	4.8	3.5	0.07 (0.06-0.07)
Major chronic comorbidities			
Diabetes	37.7	35.5	0.05 (0.04-0.05)
Heart failure	11.2	9.8	0.05 (0.05-0.05)
Ischemic heart disease	15.0	14.2	0.02 (0.02-0.03)
Stroke or transient ischemic attack	4.5	3.6	0.05 (0.05-0.05)
Chronic obstructive pulmonary disease or asthma	21.6	22.9	0.03 (0.03-0.03)
Chronic kidney disease	18.5	17.6	0.02 (0.02-0.03)
Neuropsychiatric comorbidities			
Dementia or Alzheimer disease	9.2	5.9	0.13 (0.13-0.13)
Intellectual or developmental disability	2.2	2.0	0.02 (0.02-0.02)
Depression	21.5	20.9	0.02 (0.01-0.02)
Anxiety disorder	18.6	17.9	0.02 (0.02-0.02)
Serious mental illness (bipolar disorder, schizophrenia/psychosis)	10.1	10.1	0 (0-0)

Abbreviation: NA, not applicable.

^a^ Data are expressed as mean percentages unless otherwise indicated. Available was defined as a county in which at least 1 aligned D-SNP was operating (identified using Applicable Integrated Plan indicator data).

^b^ Determined based on the county where they were living during the first month of enrollment. If the county was missing, either the county in a subsequent month or the county identified in the Social Security Administration data was used.

^c^ The approach for calculating these data was based on the methods of Austin.^33^

^d^ Included Medicare beneficiaries with at least 1 calendar year (2020 or 2021) of continuous enrollment in Medicare Parts A and B and 1 study year (2021 or 2022) with at least 6 months enrollment in a full benefit D-SNP.

^e^ The score is a percentile ranking (range, 1-100); higher values indicate greater deprivation. The score was missing for 269 722 beneficiary-years (5.5% of the total).

^f^ Based on July of the study year.

^g^ Had at least 6 months of D-SNP enrollment.

^h^ Identified from the Research Triangle Institute (RTI) race code.

^i^ Combined category from the RTI race codes of “other” and “unknown.”

^j^ Missing for 132 beneficiary-years (0.002% of the total).

^k^ Defined as 100 days or longer.

^l^ Data were available for 1 382 617 beneficiary-years.

## Discussion

In this national cohort study of full-benefit D-SNP enrollees in 2021 and 2022, we found that the availability of exclusively aligned D-SNPs and the enrollment of beneficiaries in such plans was increasing in absolute terms; however, the overall proportion enrolled remained low (this was partly because the total number of D-SNP enrollees increased substantially). More than 1 in 5 beneficiaries enrolled in fully integrated D-SNPs were in unaligned plans in 2022. More than 9 in 10 beneficiaries in highly integrated D-SNPs and all beneficiaries in coordination-only D-SNPs were in unaligned plans in 2022.

Beneficiaries residing in counties with available aligned D-SNPs had fewer social risk factors (such as lower area deprivation index scores) than beneficiaries residing in counties without available aligned D-SNPs. However, more medically complex beneficiaries were enrolled in aligned D-SNPs than in unaligned D-SNPs, likely reflecting the higher prevalence of such beneficiaries already enrolled in fully integrated D-SNPs.

These findings are noteworthy for 2 key reasons. First, they highlight the limited overall availability of exclusively aligned D-SNPs. Although policymakers are increasingly pushing to expand the availability of integrated care plans for dual-eligible beneficiaries, and are focusing on D-SNPs, the number of D-SNPs that manage Medicare and Medicaid benefits and spending for the same individuals is relatively low and geographically concentrated. Indeed, the number of dual-eligible beneficiaries enrolled in D-SNPs increased substantially from 2021 to 2022, but the geographic availability of aligned D-SNPs remained limited.

Second, a substantial number of fully integrated D-SNPs (the most integrated category of D-SNP) did not have any exclusively aligned enrollment during the study period. This raises concerns because a lack of fully aligned enrollment could erode opportunities and incentives for fully integrated D-SNPs to coordinate the full range of Medicare and Medicaid services for enrollees. Although the CMS plans to require all fully integrated D-SNPs have exclusively aligned enrollment by 2025,^9,19^ our findings imply the CMS may have difficulty accomplishing this goal. It remains unclear how this requirement would change the availability of and enrollment in fully integrated D-SNPs, and potential conversion of fully integrated D-SNPs to other types of D-SNPs such as highly integrated D-SNPs. Further actions and assistance may be needed to ensure that all fully integrated D-SNPs become exclusively aligned by 2025 and that beneficiaries do not fall through the cracks into plans with fewer tools to coordinate coverage and care.

More incentives and reforms may also be needed to encourage beneficiaries to enroll and remain in exclusively aligned plans. New CMS regulations may help increase such enrollment by establishing a continuous (monthly) open enrollment period for integrated care plans, including those with available exclusively aligned D-SNPs, while limiting the frequency of permitted enrollment into nonaligned D-SNPs.^10^

However, it is important to point out that beneficiary decisions to not enroll in or to disenroll from D-SNPs with aligned enrollment may signal dissatisfaction with coverage options and services in integrated and aligned D-SNPs. Our systematic review^14^ of integrated care plans (including D-SNPs) found that despite some positive results for fully integrated D-SNPs and for Programs of All-Inclusive Care for the Elderly in reducing nursing home use and expanding access to community-based services, much of the evidence on the effectiveness of integrated plans in coordinating care, improving outcomes, and reducing costs is limited and inconclusive.

Similarly, Mellor et al^35^ found that integrated care plans were associated with some improvements in patient experience, but such plans were not associated with any differences in care coordination and access. To the extent that exclusively aligned D-SNPs are an even more integrated form of fully integrated D-SNPs, which show the best evidence to date, and to the extent that it is the degree of integration in fully integrated D-SNPs that is beneficial, it is possible that aligned fully integrated D-SNPs may lead to better outcomes than unaligned D-SNPs. However, much remains unknown. More data are needed to better understand the experiences of dual-eligible beneficiaries enrolled in aligned D-SNPs to identify where tailored efforts are needed to improve how these plans serve enrollees, particularly in socially disadvantaged communities.^36,37^

Our findings also imply that further reforms may be needed to promote exclusively aligned enrollment in highly integrated D-SNPs, which currently have limited alignment status and enrollment. However, it is important to note that there is a lack of evidence on the effectiveness of highly integrated D-SNPs to date because these plans have only been in existence since 2021. As a result, it may be premature to invest more resources in enrolling more dual-eligible beneficiaries in highly integrated D-SNPs, particularly if they would be better off in fully integrated D-SNPs, which have better evidence to date.^14^ This again raises concerns about what will happen to the 1 in 5 fully integrated D-SNP beneficiaries whose plans were not exclusively aligned if these D-SNPs are unable to achieve the aligned designation from the CMS by 2025. Policymakers may need to allow existing fully integrated D-SNPs more time to become aligned and invest more federal and state resources for the expansion of these plans to make them available to more beneficiaries.

### Limitations

This study has several limitations. First, our focus was on enrollment in exclusively aligned D-SNPs (defined as a plan-level characteristic by the CMS). Some beneficiaries in unaligned D-SNPs may have, nonetheless, belonged to Medicare and Medicaid plans operated by the same parent organization even if the D-SNP to which they belonged did not have 100% exclusively aligned enrollment as required by the CMS. Relatedly, we relied solely on Medicare plan data, which means that we could not observe specific plan carve outs that exist solely in Medicaid data.

Second, we examined the first 2 years (2021 and 2022) that the exclusive alignment designation was made available to D-SNPs by the CMS. The composition of beneficiaries enrolled in these plans will likely change.

Third, this was a descriptive study. Any observed differences between the aligned D-SNPs and the unaligned D-SNPs should not be taken as causal relationships. There is a need for research to assess the effectiveness of aligned plans in improving outcomes and reducing costs among dual-eligible beneficiaries.

## Conclusions

This study found that availability of and enrollment in D-SNPs with exclusively aligned enrollment are increasing, but the overall proportion enrolled remains low. Further reforms are needed to promote aligned enrollment.

## Notes

Supplement 1. **eFigure 1.** Study Population Selection Flowchart **eFigure 2.** Dual Special Needs Plans by Plan Type and Alignment Status, 2021 and 2022 **eTable.** Baseline (Prior Year) Characteristics for Full-Benefit Dual-Eligible Beneficiaries in 2021-2022, by Residence in Counties with vs without Aligned Plan Offerings, Excluding Health Risk Assessment Claims and Medicare Advantage Beneficiaries Not on Reliable Contracts at Baseline

Supplement 2.
Data sharing statement
