# Supplementary material for: Nationwide Availability of and Enrollment in Medicare and Medicaid Dual-Eligible Special Needs Plans With Exclusively Aligned Enrollment
Source: JAMA Health Forum. 2024 Oct 18;5(10):e243546. doi: 10.1001/jamahealthforum.2024.3546 (PMC11581671; doi:10.1001/jamahealthforum.2024.3546)
Supplement: Supplement 1. — eFigure 1. Study Population Selection Flowchart eFigure 2. Dual Special Needs Plans by Plan Type and Alignment Status, 2021 and 2022 eTable. Baseline (Prior Year) Characteristics for Full-Benefit Dual-Eligible Beneficiaries in 2021-2022, by Residence in Counties with vs without Aligned Plan Offerings, Excluding Health Risk Assessment Claims and Medicare Advantage Beneficiaries Not on Reliable Contracts at Baseline [file jamahealthforum-e243546-s001.pdf]

Supplemental Online Content

Johnston KJ, Hendricks M, Dabas M, et al. Nationwide availability of and enrollment in Medicare and Medicaid dual-eligible special needs plans with exclusively aligned enrollment. *JAMA Health Forum*. Published online October 18, 2024. doi:10.1001/jamahealthforum.2024.3546

eFigure 1. Study Population Selection Flowchart

eFigure 2. Dual Special Needs Plans by Plan Type and Alignment Status, 2021 and 2022

eTable. Baseline (Prior Year) Characteristics for Full-Benefit Dual-Eligible Beneficiaries in 2021-2022, by Residence in Counties with vs without Aligned Plan Offerings, Excluding Health Risk Assessment Claims and Medicare Advantage Beneficiaries Not on Reliable Contracts at Baseline

This supplemental material has been provided by the authors to give readers additional information about their work.

© 2024 Johnston et al. *JAMA Health Forum*.

eFigure 1. Study Population Selection Flowchart

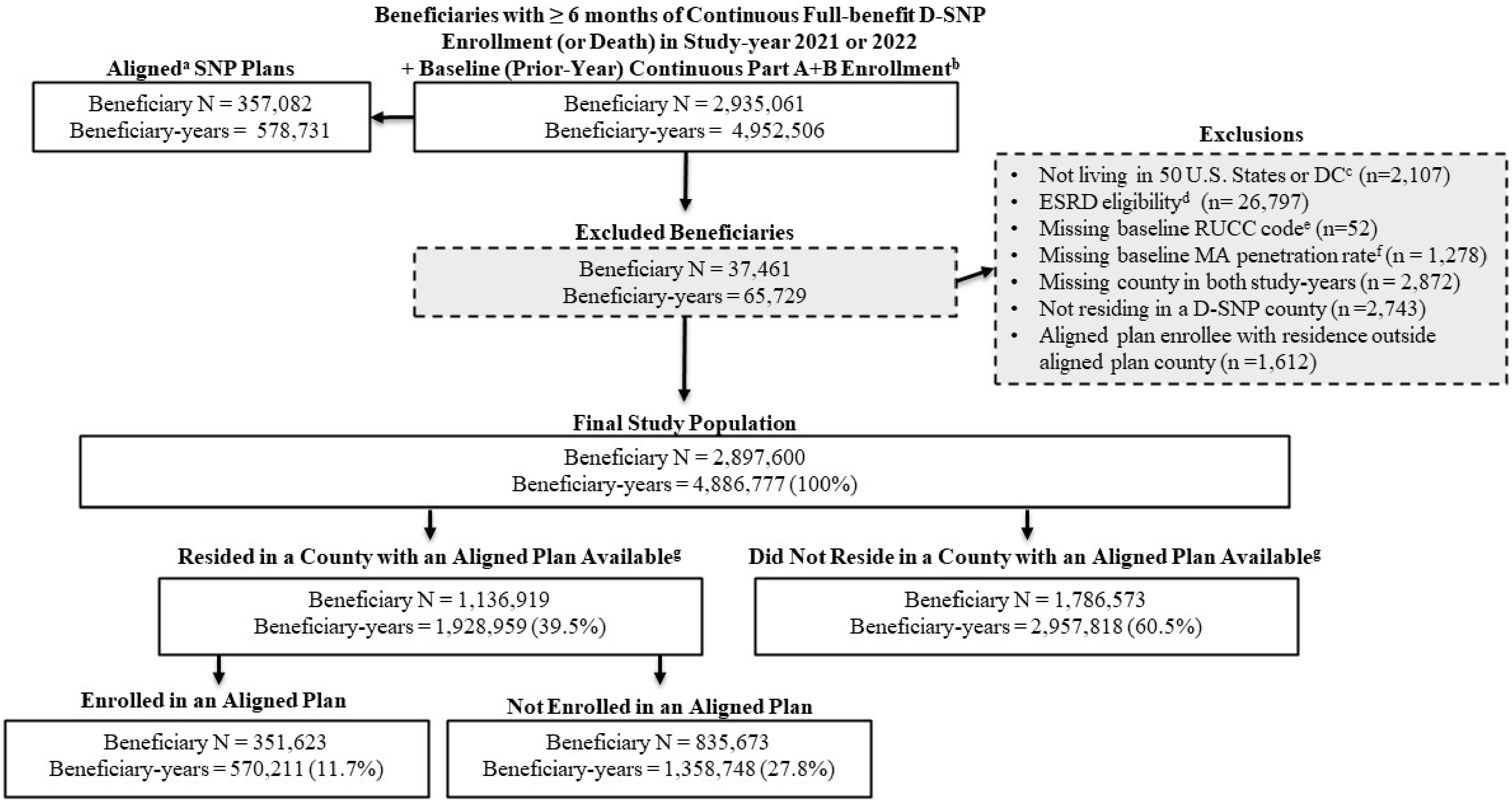

Abbreviations: N, number; RUCC, rural-urban commuting area codes; MA, Medicare Advantage; TM, traditional Medicare; ESRD, end-stage renal disease.
[a]Aligned DSNP plans were identified using the Applicable Integrated Plan (AIP) indicator data published by the Centers for Medicare & Medicaid Services (https://www.cms.gov/medicaid-chip/medicare-coordination/qualified-beneficiary-program/d-snps-integration-unified-appeals-grievance-requirements) and linked by unique 5-digit contract and 3-digit plan identifiers to SNP plan type indicators in the SNP data file (https://www.cms.gov/data-research/statistics-trends-and-reports/medicare-advantagepart-d-contract-and-enrollment-data/special-needs-plan-snp-data) and to the Part C Contract Number and the Part C PBP Number in the Master Beneficiary Summary File (MBSF). A single Contract/PBP Number combination operating in two separate states with two separate D-SNP plan types (one coordination only dual eligible SNP and one highly integrated dual eligible SNP) was treated as two separate plans.

© 2024 Johnston et al. *JAMA Health Forum*.

[b]Medicare beneficiaries with at least 1-calendar year (2020 or 2021) of continuous baseline enrollment in Medicare Parts A+B (TM or MA) and a following study-year (2021 or 2022) of at least six months of full benefit dual eligible special needs plan enrollment. Excluding ESRD beneficiaries, those residing outside of 50 U.S. states and DC), and those missing baseline MA penetration rates or Rural-Urban Continuum Codes (RUCC). Individuals who died in the first six months of the study year were included if they met the criteria up to the month of their death. Beneficiaries were assigned to the plan that they had for the most months during the study year. If two plans had the same number of months, then beneficiaries were assigned to the last plan they had during the study year.

[c]Individuals must have been living in the 50 U.S. states or DC in both the study year and the baseline year based on state social security administration (SSA) and Federal Information Processing Standards (FIPS) codes in the Master Beneficiary Summary File. Individuals in Puerto Rico-based D-SNPs in the study year were also excluded.

[d]Original or current ESRD eligibility in the study year

[e]2023 RUCCs from the USDA Economic Research Service in the US Department of Agriculture.

[f]Baseline MA state/county penetration rates are based on the July files located here: https://www.cms.gov/data-research/statistics-trends-and-reports/medicare-advantagepart-d-contract-and-enrollment-data/ma-state/county-penetration

[g]Counties with aligned plans were defined as counties in which at least aligned one plan was operating. Beneficiaries were living in a county with an aligned plan if the county they were living in during the first month they were enrolled in the dual special needs plan included at least one aligned plan. For observations where beneficiaries' county was missing on their first enrollment month, we used either the county in a subsequent month or the county identified in the social security administration state and county code for the study year. Beneficiary-years in which the beneficiary was on an aligned plan or D-SNP but the beneficiary did not reside in a county with an aligned plan or D-SNP were excluded.

© 2024 Johnston et al. *JAMA Health Forum*.

eFigure 2. Dual Special Needs Plans by Plan Type and Alignment Status, 2021 and 2022

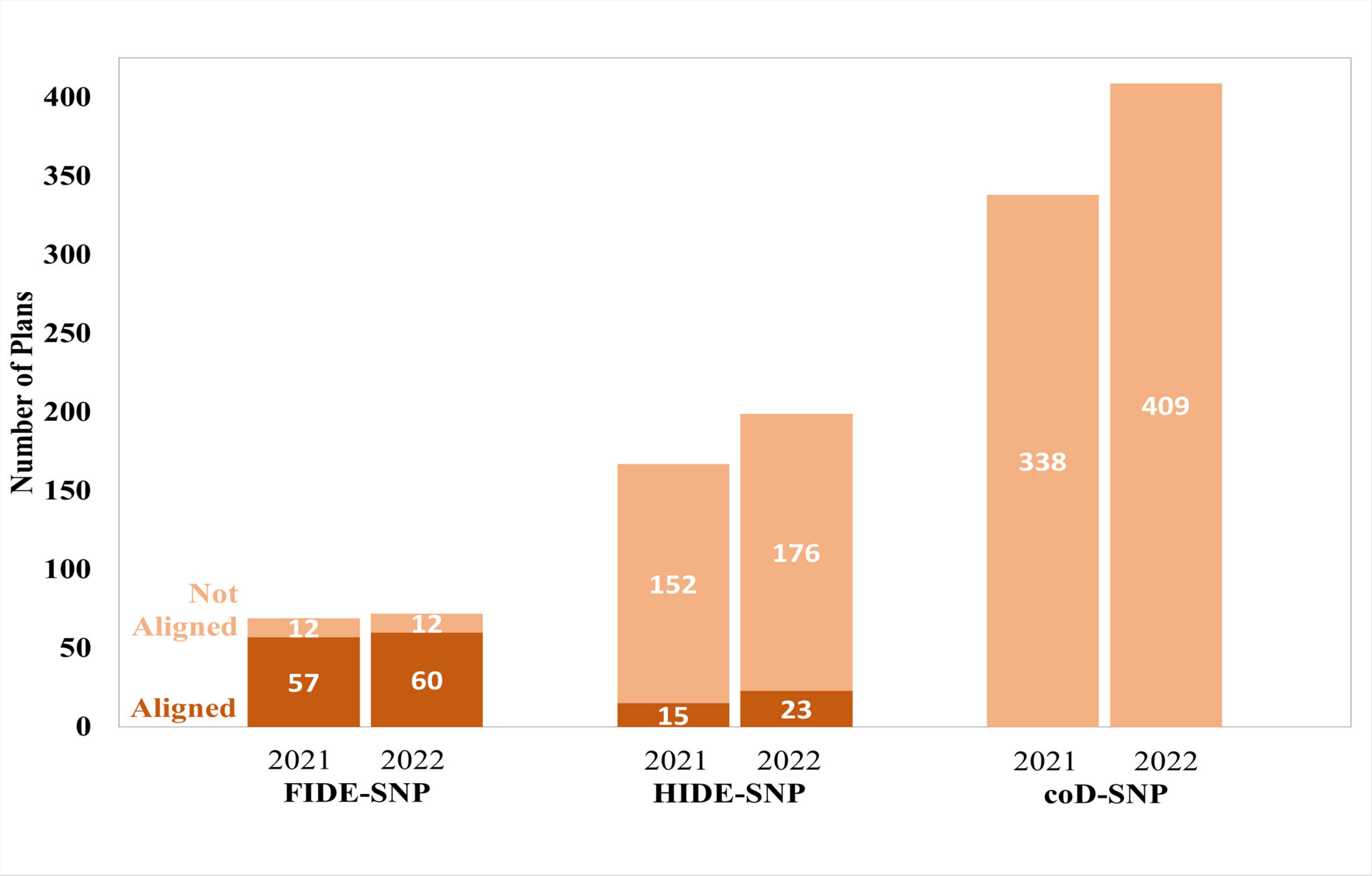

Abbreviations: FIDE-SNP, fully integrated dual eligible special needs plan; HIDE-SNP, highly integrated dual eligible special needs plan; coD-SNP, coordination only dual eligible special needs plan.

Notes: Aligned D-SNP plans were identified using the Applicable Integrated Plan (AIP) indicator data published by CMS (https://www.cms.gov/medicaid-chip/medicare-coordination/qualified-beneficiary-program/d-snps-integration-unified-appeals-grievance-requirements) and linked by unique 5-digit contract and 3-digit plan identifiers to SNP plan type

© 2024 Johnston et al. *JAMA Health Forum*.

indicators in the SNP data file (https://www.cms.gov/data-research/statistics-trends-and-reports/medicare-advantagepart-d-contract-and-enrollment-data/special-needs-plan-snp-data) and to the Part C Contract Number and the Part C PBP Number in the Master Beneficiary Summary File (MBSF). A single Contract/PBP Number combination operating in two separate states with two separate D-SNP plan types (one coordination only dual eligible SNP and one highly integrated dual eligible SNP) was treated as two separate plans.

© 2024 Johnston et al. *JAMA Health Forum*.

eTable. Baseline (Prior Year) Characteristics for Full-Benefit Dual-Eligible Beneficiaries in 2021-2022, by Residence in Counties with vs without Aligned Plan Offerings, Excluding Health Risk Assessment Claims and Medicare Advantage Beneficiaries Not on Reliable Contracts at Baseline

	Non-residence in County with Aligned Plan(s)[b,c]	Residence in County with Aligned Plan(s)[b,c]	SMD[d]
**Beneficiary-years**	**2,775,237**	**1,838,165**	**-**
**Beneficiaries, N[a]**	**1,712,713**	**1,096,861**	**-**
**Baseline Local Area Characteristics**			
Area Deprivation Index[e], mean (SD)	66.81 (26.36)	43.15 (29.10)	0.85
Rural or Micropolitan[f]	22.1%	7.9%	0.41
Medicare Advantage County Penetration Rate[g]	45.1%	48.0%	0.23
**Study Year Insurance Enrollment**			
Total Enrollment Months, mean (SD)	11.75 (1.37)	11.76 (1.37)	0.00
Highly Integrated Dual Eligible SNP[h]	26.0%	48.4%	0.48
Fully Integrated Dual Eligible SNP[h]	3.6%	25.6%	0.65
Coordination Only Dual Eligible SNP[h]	70.3%	26.0%	0.99
Current Medicare Entitlement Status			
Age ≥65	55.4%	72.6%	0.36
Disability	44.5%	27.4%	0.36
**Baseline Demographic Characteristics**			
Age at End of Baseline, mean (SD)	63.58 (14.30)	68.27 (13.76)	0.34
Sex			
Female	63.1%	62.8%	0.01
Male	36.9%	37.2%	0.01
Race and Ethnicity[i]			
American Indian/Alaska Native	0.8%	0.3%	0.07
Asian/Pacific Islander	6.0%	12.4%	0.22
Black (or African American)	27.5%	21.2%	0.15
Hispanic	15.3%	33.7%	0.44
Non-Hispanic White	48.8%	29.6%	0.40
Other / Unknown[j]	1.7%	2.8%	0.08
**Baseline Health Status**			
CMS-HCC Risk Score, mean (SD)[k]	1.56 (1.47)	1.53 (1.37)	0.02
Long Term Care Institutionalization[l]	1.8%	1.9%	0.01
Frailty Level[m] (Community-Dwelling Ages ≥65)			
Robust	34.5%	37.1%	0.05
Prefrail	47.7%	47.1%	0.01
Mild frailty	13.9%	12.3%	0.05
Moderate to severe frailty	4.0%	3.6%	0.02
Major Chronic Comorbidities[n]			
Diabetes	34.7%	38.1%	0.07
Heart Failure	11.3%	11.2%	0.00
Ischemic Heart Disease	14.4%	15.4%	0.03
Stroke/Transient Ischemic Attack	3.9%	4.3%	0.02
COPD / Asthma	26.0%	23.3%	0.06
Chronic Kidney Disease	16.6%	19.2%	0.07
Neuropsychiatric Comorbidities[o]			
Alzheimer's Disease / Dementia	4.4%	6.7%	0.10
Intellectual/Developmental Disability	3.3%	2.1%	0.08
Depression	22.6%	21.9%	0.02
Anxiety Disorder	23.7%	19.0%	0.12
Serious Mental Illness (Bipolar/Psychoses)	13.5%	10.5%	0.09

© 2024 Johnston et al. *JAMA Health Forum*.

Abbreviations: FIDE, fully integrated dual eligible; HIDE, highly integrated dual eligible; N, number; SMD, standardized mean difference; SD, standard deviation; D-SNP, dual eligible special needs plan; SNP, special needs plan; CMS-HCC; Centers for Medicare and Medicaid Services Hierarchical Condition Categories; COPD, Chronic Obstructive Pulmonary Disease.

Notes: We removed health risk assessment (HRA) records from baseline MA and TM claims and recalculated the baseline variables that were dependent upon claims following the method used by Jung et al. (2023). We excluded carrier or outpatient claims as HRA claims if the claim had a HCPCS code of G0438, G0439, G0402, or was a home visit carrier claim with a HCPCS code of 99341, 99342, 99343, 99344, 99345, 99347, 99348, 99348, 99350 and was the only home visit for the individual in the year.

To identify reliable MA contracts, we applied the methods of Jung et al. (2022) to validate all MA encounter data for each contract against Medicare Analysis and Review (MedPAR) to remove contracts with a high level of missingness. Because HEDIS measures were not available for 2021-2022 contracts, we validated the encounter files against MedPAR data. Reliable contracts were defined as those with least 2,500 enrollees and less than 10% missing hospitalizations. Beneficiaries that were not on reliable contracts for all 12 months of the baseline year were removed from the analysis.

[a]Medicare beneficiaries with at least 1-calendar year (2020 or 2021) of continuous baseline enrollment in Medicare Parts A+B (Traditional Medicare, TM or Medicare Advantage, MA) and a following study-year (2021 or 2022) of at least six months of full benefit dual eligible special needs plan enrollment, including Medicare Parts A+B enrollment (TM or MA). Excluding end-stage renal disease beneficiaries, those living outside of the U.S. (50 states and the District of Columbia), and those missing baseline Medicare Advantage County penetration rates or Rural-Urban Continuum Codes (RUCC). Individuals who died in the first six months of the study year were included if they met the criteria up to the month of their death.

[b]Aligned D-SNP plans were identified using the Applicable Integrated Plan (AIP) indicator data published by CMS (https://www.cms.gov/medicaid-chip/medicare-coordination/qualified-beneficiary-program/d-snps-integration-unified-appeals-grievance-requirements) and linked by unique 5-digit contract and 3-digit plan identifiers to SNP plan type indicators in the SNP data file (https://www.cms.gov/data-research/statistics-trends-and-reports/medicare-advantagepart-d-contract-and-enrollment-data/special-needs-plan-snp-data) and to the Part C Contract Number and the Part C PBP Number in the Master Beneficiary Summary File (MBSF). A single Contract/PBP Number combination operating in two separate states with two separate D-SNP plan types (one coordination only dual eligible SNP and one highly integrated dual eligible SNP) was treated as two separate plans.

[c]Counties with aligned plans were defined as counties in which at least aligned one plan was operating. Beneficiaries were living in a county with an aligned plan if the county they were living in during the first month they were enrolled in the dual special needs plan included at least one aligned plan. For observations where beneficiaries' county was missing on their first enrollment month, we used either the county in a subsequent month or the county identified in the social security administration state and county code for the study year. Beneficiary-years in which the beneficiary was on an aligned plan or D-SNP but the beneficiary did not reside in a county with an aligned plan or D-SNP were excluded.

[d]The approach for calculating SMDs for continuous and categorical variables was drawn from Austin (2009), here: https://pubmed.ncbi.nlm.nih.gov/19757444/. We calculated a separate SMD for each level of multi-level categorical variables using the approach for dichotomous variables described in Austin (2009).

[e]We linked Area Deprivation Index (ADI) scores to beneficiaries using 9-digit zip codes. The ADI is scored as a percentile ranking ranging from 1 to 100, where higher values indicate greater deprivation. The ADI was missing for 269,722 beneficiary-years (5.5% of total beneficiary-years).

[f]The mean percentage of beneficiary-years (2021-2022) during which beneficiaries were categorized as living in either metropolitan or rural/micropolitan counties. Using the 2023 RUCC from the USDA Economic Research Service in the US Department of Agriculture, counties with RUCC codes of 1-3 were categorized as metropolitan and those with RUCC codes of 4-9 as rural and metropolitan.

[g]Medicare Advantage state/county penetration for July of the study year was retrieved from here: https://www.cms.gov/data-research/statistics-trends-and-reports/medicare-advantagepart-d-contract-and-enrollment-data/ma-state/county-penetration

[h]We only included individuals with at least 6 months of D-SNP enrollment in the study year (or enrollment up until death for individuals who died in the first 6 months of the year) in the cohort. We assigned beneficiaries to the plan that they had for the most months during the study year. If two plans had the same number of months, then we assigned beneficiaries to the last plan they had during the study year.

[i]Race and ethnicity were identified from the Research Triangle Institute race code, which was created by taking the beneficiary race code used by the Social Security Administration and applying an algorithm that uses first and last name to identify more beneficiaries as Hispanic or Asian.

[j]The "Other" and "Unknown" categories of the Research Triangle Institute race code were combined to create the "Other/Unknown" category.

© 2024 Johnston et al. *JAMA Health Forum*.

[k]We used version 28 of the CMS HCC risk score. The CMS-HCC risk score was missing for 132 beneficiary-years (0.002% of total beneficiary-years).

[l]Long term care institutionalization is defined as one hundred or more days in long term care. We obtained long term care days from the Minimum Data Set (MDS) 3.0.

[m]We calculated frailty scores only for non-institutionalized individuals aged sixty-five and older. The frailty score was available for 1,382,617 beneficiary-years.

[n]To identify major chronic comorbidities, we applied the 30 CCW Chronic Condition algorithms to baseline year TM and MA claims (with no lookback). The COPD and Asthma algorithms were combined to create one category.

[o]To identify neuropsychiatric comorbidities, we applied either the 30 CCW Chronic Condition (for Alzheimer's Disease and Related Dementias) or the Other Chronic Health, Mental Health, and Potentially Disabling Conditions algorithms to baseline year TM and MA claims (with no lookback). Autism Spectrum Disorder, Cerebral Palsy, and Intellectual Disability and Related Conditions algorithms were combined to create the "Intellectual and Developmental Disability" category. The Bipolar Disorder and Schizophrenia and Other Psychotic Disorders algorithms were combined to create the "Serious Mental Illness" category. The Alzheimer's Disease and Non-Alzheimer's Dementia algorithms were combined to create the "Alzheimer's Disease and Related Dementias" category.

© 2024 Johnston et al. *JAMA Health Forum*.
