# Supplementary material for: Nationwide Availability of and Enrollment in Medicare and Medicaid Dual-Eligible Special Needs Plans With Exclusively Aligned Enrollment
Source: JAMA Health Forum. 2024 Oct 18;5(10):e243546. doi: 10.1001/jamahealthforum.2024.3546 (PMC11581671; doi:10.1001/jamahealthforum.2024.3546)
Supplement: Supplement 2. — Data sharing statement [file jamahealthforum-e243546-s002.pdf]

Data Sharing Statement

Johnston. Nationwide Availability of and Enrollment in Medicare and Medicaid Dual-Eligible Special Needs Plans With Exclusively Aligned Enrollment. *JAMA Health Forum*. Published October 18, 2024. doi:10.1001/jamahealthforum.2024.3546

Data
Data available: No

Additional Information
Explanation for why data not available: This study used research identifiable patient data on the Medicare Chronic Condition Data Warehouse (CCW) that is only obtainable via execution of a data use agreement with the Centers for Medicare and Medicaid Services (CMS).
